# Overexpression of hepatoma-derived growth factor in melanocytes does not lead to oncogenic transformation

**DOI:** 10.1186/1471-2407-11-457

**Published:** 2011-10-20

**Authors:** Angela Sedlmaier, Nicolas Wernert, Rainer Gallitzendörfer, Mekky M Abouzied, Volkmar Gieselmann, Sebastian Franken

**Affiliations:** 1 Institute of Biochemistry and Molecular Biology, University of Bonn, Nussallee 11, 53115 Bonn, Germany; 2 Institute of Pathology, University Hospital Bonn, Bonn, Germany; 3 Faculty of Pharmacy, University of El-Minia, Minia, Egypt

## Abstract

**Background:**

HDGF is a growth factor which is overexpressed in a wide range of tumors. Importantly, expression levels were identified as a prognostic marker in some types of cancer such as melanoma.

**Methods:**

To investigate the presumed oncogenic/transforming capacity of HDGF, we generated transgenic mice overexpressing HDGF in melanocytes. These mice were bred with mice heterozygous for a defective copy of the Ink4a tumor suppressor gene and were exposed to UV light to increase the risk for tumor development both genetically and physiochemically. Mice were analyzed by immunohistochemistry and Western blotting. Furthermore, primary melanocytes were isolated from different strains created.

**Results:**

Transgenic animals overexpressed HDGF in hair follicle melanocytes. Interestingly, primary melanocytes isolated from transgenic animals were not able to differentiate *in vitro *whereas cells isolated from wild type and HDGF-deficient animals were. Although, HDGF^-/-^/Ink4a^+/- ^mice displayed an increased number of epidermoid cysts after exposure to UV light, no melanomas or premelanocytic alterations could be detected in this mouse model.

**Conclusions:**

The results therefore provide no evidence that HDGF has a transforming capacity in tumor development. Our results in combination with previous findings point to a possible role in cell differentiation and suggest that HDGF promotes tumor progression after secondary upregulation and may represent another protein fitting into the concept of non-oncogene addiction of tumor tissue.

## Background

HDGF is a heparin-binding growth factor originally isolated from conditioned medium of HuH-7 cells [1,2]. Several studies reported that HDGF promotes proliferation, differentiation, and migration of several cell types, such as vascular smooth muscle cells, when applied exogeneously to the culture medium or when overexpressed endogeneously [3-6]. Studies revealed that HDGF is expressed in most organs (liver, brain, lung, intestine etc.) both during embryonic development and throughout the organism's life [7]. Together with its mitogenic activity HDGF was therefore implicated in organ development and tissue differentiation [5,8,9]. Models of vascular injury and ulcerative colitis pointed towards a function of HDGF in tissue repair [10-12]. This suggestion was supported by the classification of HDGF as an alarmin, a protein signaling cell and tissue damage to the immune system [13,14]. Surprisingly, HDGF-knockout (HDGF^-/-^) mice are viable and show no developmental phenotype [15]. Over the last years, the overexpression of HDGF in various tumors attracted more and more attention. Up to now HDGF has been reported to be overexpressed in non-small cellular lung cancer [16], hepatocellular carcinoma [17,18], colorectal carcinoma [19,20], oesophagal carcinoma [21], pancreatic carcinoma [22] and melanoma [23]. Furthermore, a correlation between the degree of HDGF overexpression and the course of disease was found for some types of cancer, including melanoma, leading to the classification of HDGF as a novel prognostic marker [16,18,20-22,24]. In addition, several studies suggested a role for HDGF in tumor angiogenesis [25,26], metastasis [19,26], and apoptosis [27,28]. So far, the influence of HDGF on tumors was studied by si-RNA mediated or antibody-mediated reduction of HDGF in tumor mouse models. The accordant results showed, that a reduction of HDGF in tumor cells resulted in the formation of smaller tumors, reduced tumor angiogenic and metastatic capacity, and an improved response to chemotherapeutic treatment [25,26]. However, a direct influence of an initial overexpression of HDGF has so far not been investigated.

In this study, we present for the first time results on the presumed putative oncogenic/transforming capacity of this mitogenic growth factor by studying the influence of HDGF on tumor development in a transgenic mouse model overexpressing HDGF in melanocytes, a cell type from which malignant melanoma, a highly aggressive type of cancer, arises. This model was generated to study the influence of HDGF-overexpression in a non-transformed cell type *in vivo*.

## Methods

### Construction of the Tyrosinase(Tyr)-HDGFgene construct

The Tyr-HDGFgene construct consists of the complete HDGF genomic sequence, except for intronI which was shortened from 6, 377 bp to 786 bp. The shortened HDGF gene was cloned into the phs3.6 Tyrosinase-lacZ(-6.1) vector (kindly provided by Friedrich Beermann, IRES, Epalinges, Switzerland) in multiple steps. First, the lacZ gene was removed by incubating the vector with the restriction enzymes SmaI and NotI (Fermentas, St. Leon-Rot, Germany). Before the plasmid was allowed to religate, the ends were blunted by using the Klenow Fragment (Fermentas, St. Leon-Rot, Germany). In a second step, this plasmid was linearized through incubation with XhoI and ligated with the compatible ends of the SalI incubated shortened HDGF gene.

### Generation of Transgenic Mice

The HDGF^Tyr^-transgenic mice were generated through injection of the Tyr-HDGFgene construct into the pronucleus of fertilized eggs. The genotype of the emanated mice and their offspring was determined by PCR on DNA from tail biopsies. The following primers were used to distinguish the Tyr-HDGFgene construct from the wildtype HDGF gene: mHDGFgeneConstruct_sense, 5'-GTATCGATAAGCTTGGTACCATG-3' and HDGFwt-antisense, 5'-CTTGGTATTTGTTGGC TGTTGA-3'. PCR conditions were as follows: denaturation for 3 min at 95°C, followed by 35 cycles of 30 sec at 95°C, 60 sec at 54°C and 60 sec at 72°C. This reaction resulted only in a fragment of 943 bp if the Tyr-HDGFgene construct was present in the sample DNA. All mice were backcrossed for more than ten generations into the C57BL6N background. Furthermore, the following mouse lines were generated by backcrossing: HDGF^Tyr^/HDGF^-/-^, HDGF^Tyr^/Ink4a^-/-^, HDGF^-/-^/Ink4a^-/-^. The HDGF^-/- ^mouse line was generated by Rainer Gallitzendörfer (IBMB, University of Bonn, Germany) [15], whereas the Ink4a^-/- ^mice were kindly provided by Clemens Schmitt (Charité - University Berlin, Germany) with permission of Manuel Serrano (Spanish National Cancer Research Centre, Madrid, Spain) [29].

Mice used in this study were housed under standard conditions in a 12 hour light-dark cycle and provided with food and water *ad libitum*. All experiments were carried out in accordance with local and state regulations for animal research.

### Southern Blot Analysis

Genomic DNA was isolated from mouse tails by phenol/chloroform extraction. 10 μg DNA was fragmented by incubation with the restriction enzymes BglII and HindIII (Fermentas, St. Leon-Rot, Germany), separated on a 0.8% agarose gel and blotted onto a Hybond™-N^+ ^nylon filter (Amersham, GE Healthcare, Munich, Germany) by performing a capillary blot. The probe spanning exonIV, intronIV and exonV of the HDGF gene was labeled with [α^32^P]-dCTP by using the Megaprime-DNA labeling Kit^® ^(Amersham, GE Healthcare, Munich, Germany) according to manufacturer's instruction. Hybridization was carried out by incubating the membrane in FBI-buffer (SSPE buffer, 10% PEG-8000, 7% SDS) containing the radioactive labeled probe (1 × 10^6 ^cpm per cm^2 ^filter). Signals were detected by exposing the filters to films (Kodak, Stuttgart, Germany) for 24 hours.

### UVB-Irradiation of newborn mice

Mice of 1-3 days of age were placed on a sterile cotton sheet and irradiated with a single UVB-dose of 6.14 kJ/m^2^. The light pannel contained four TL/12 UVB-light bulbs. The dose was determinded by a scanning spectrophotometer. The following genotypes were used for this experiment: HDGF^Tyr^/INK4a^+/-^, Ink4a^+/-^, HDGF^-/-^/INK4a^+/-^, HDGF^Tyr^, wildtype and HDGF^-/-^.

### Isolation of total RNA and RT-PCR

Total RNA was isolated using Trizol (Invitrogen), following the manufacturer's instructions. cDNA was synthesized from 1 μg total RNA using QuantiTect Reverse Transcription Kit (Qiagen) according to the manual. For the detection of HDGF-cDNA a PCR with primer HDGF-sense (5'GCGGCGGTACCATGTCGCGATCCAACCGG3') and HDGF-antisense (5' GAGACTCGAGT GAGTGAGGGAGTAG3'), and REDTaq^® ^ReadyMix^™ ^PCR Reaction Mix (Sigma) was performed following the manufacturer's instructions. To specifically amplify the HDGF-cDNA expressed from the Tyr-HDGFgene construct the primers mHDGFgeneConstruct_sense (5'GTATCGATAAGCTTGGTACCATG3') and HDGF-antisense (5'GAGACTCGAGTGAGTGAGGGAGTAG3') were used. PCR conditions were as follows: denaturation for 3 min at 95°C, followed by 35 cycles of 30 sec at 95°C, 60 sec at 56°C and 60 sec at 72°C.

### Immunostaining and Histology

Tissue sections were prepared from skin samples from each group of mice and for each treatment. Tissues were fixed at 4°C over night (4% buffered formalin/10% sucrose), paraffin embedded and cut into 4 μm sections. HE staining was performed as previously described [30]. Sections were deparaffinized in 10 mM Citrate-buffer, pH 6.0 and blocked with 3% BSA/TBS. Affinity-purified polyclonal rabbit-anti-mouse HDGF (dilution 1:100 in 1% BSA/TBS) antibodies (Abouzied et al., 2004) were used to stain mouse tissue. Control slides were incubated with 1% NGS/TBS (normal goat serum (Abcam) in TBS) without primary antibody. For immunofluorescent detection of HDGF, goat-anti-rabbit-Cy3 was used as a secondary antibody. Nuclei were stained with DAPI (Sigma). Images were aquired on an Axioskop 2 MOT (Carl Zeiss, Jena, Germany) microscope equipped with an Olympus XL-50 (Olympus, Hamburg, Germany) digital camera.

### Bleaching of pigmented Tissue sections

Bleaching of tissue sections was carried out after deparaffinization in a solution consisting of 55% benzyl alcohol, 25% acetone, 15% H_2_O_2 _(10%) and 5% ammonium hydroxide solution (25%). According to the grade of pigmentation incubation time ranged from 15-120 min at 37°C.

### *In Situ *Hybridisation

For the detection of tyrosinase mRNA a tyrosinase sense (Tyr_s - control) and antisense (Tyr_as - experimental) probe were generated from C57BL6 skin cDNA (Primer: Tyr_sense 5'-AGTCTGCAG TACTCGAGTGTTTTGTATTGCCTTCTGTGGA-3'; Tyr_antisense 5'- TGAAGCTTACCCATT GTTCATTTGGCCAT-3') and transcribed on a T7-based plasmid using a digoxigenin (DIG) RNA-labeling kit (Roche Applied Science, Manheim, Germany) according to the manufacturer's manual. Four micrometer sections were cut from paraffine embedded skin samples and adhered to glass slides (Superfrost Ultra Plus, Thermo Fisher Scientific, Waltman, MA). *In situ *hybridization was performed as previously described by Fewou and coworkers [31]. Briefly, after rehydration, sections were post-fixed in 4% paraformaldehyde/PBS, before endogenous peroxidases were inhibited by incubation with 1% H_2_O_2_/PBS for 15 min. Thereafter, sections were treated with proteinase K (10 μg/ml; 4 min) and permeabilized with 0.25% Triton X-100 (4 min). Afterwards, the slices were incubated with 0.2 M HCl (8 min), 0.1 M triethanolamine/2.5 μl/ml acetic anhydride (10 min), and washed for 10 min with 2 × SSC (0.15 M NaCl/0.015 M sodium citrate) at 50°C. DIG-labeled probes (diluted 1:1, 000 in 50% (v/v) formamide, 1% Denhardt's solution, 0.2% SDS, and 10% (w/v) hybridization salt (3 M NaCl, 0.1 M PIPES and 0.1 M EDTA)) were allowed to hybridize to tissue-mRNA over night at 70°C. After removal of the hybridization solution, sections were washed with 2 × SSC at 60°C, for 20 min with 50% formamide/1 × SSC at 60°C and for 45 min with 0.1 × SSC at 70°C. DIG-labeled probes were detected by incubation with alkaline phosphatase-conjugated anti-DIG F_ab _fragment (Roche Applied Science, Manheim, Germany). Bound antibodies were further visualized by incubating the slides with BMPurple™ (Roche Applied Science, Manheim, Germany) for 7-10 days. Color development was stopped by washing with ddH_2_O, before embedding the slides with Mowiol. Images were aquired on an Axioskop 2 MOT (Carl Zeiss, Jena, Germany) microscope equipped with an Olympus XL-50 (Olympus, Hamburg, Germany) digital camera.

### Primary Melanocyte Culture

1-3 day old mice were used for the isolation of melanocytes. Neonatal mice were decapitated and desinfected, before the abdominal skin was drawn off. The whole skin was incubated overnight in dispase II-solution (5 mg/ml; 4°C; slightly shaking) before the epidermis could be separated from the dermis. Dissociation of the epidermal cells was achieved by incubation of the epidermis floating on 1 ml trypsin solution (0.025% trypsin/0.02% EDTA/PBS; 5 min; room temperature) with the stratum corneum facing up. The reaction was stopped by adding 3 ml of FCS containing medium and the epidermal cells could subsequently be detached by gentle scraping with a spattle. The cell suspension was sedimented by centrifugation (600 × g; 5 min; room temperature), before the cells were resuspended in fresh melanocyte medium (RPMI Gibco BRL including 5% FCS (Invitrogen), 2 mM Glutamax™ (Gibco BRL), 100 IU penicillin (Gibco BRL), 100 μg streptomycin (Gibco BRL), 4 μg/ml insulin (Sigma), 100 U/ml catalase (Sigma), 10 μg/ml BPE (Sigma)) and seeded into a 10 cm cell culture dish. After four days the keratinocytes made up a confluent cell layer on which the melanocytes grew. The melanocytes were detached from the keratinocytes by trypsinization for 1 min. The cells were sedimented and resuspended in melanocyte medium supplemented with 23 ng/ml 3-isobutyl-1-methylxanthen (IBMX), 100 ng/ml 12-O-tetradecanoyl-phorbol-13-acetate (TPA). Medium was replaced every three days.

### Western Blot Analysis

Tissue samples and cell pellets were lysed in HEPES buffer (20 mM HEPES/OH pH 7.4, 250 mM sucrose, 10 mM KCl, 1.5 mM MgCl_2_, 1 mM EDTA, 1 mM EGTA, 1 mM PMSF, 1 mM DTT, 50 μg/ml DNaseI, 0.1% SDS (v/v), 1 μg/ml leupeptin, 1 μg/ml pepstatin). The lysates were incubated on ice for 20 minutes followed by centrifugation at 18, 000 × g and 4°C for 20 min. The protein concentration of the supernatant was determined by using the Bio-Rad DC protein assay system (Bio-Rad Laboratories, Munich, Germany) according to the manufacturer's instructions. Soluble homogenates (40 μg total protein) were separated under denaturating conditions in 10% sodium dodecyl sulfate-polyacrylamide gels by electrophoresis and transferred onto pvdf-membranes (BioRad, Munich, Germany) using semidry blotting [32]. Membranes were stained with Coomassie solution (Coomassie Brilliant Blue R, Serva 35051) to assess equal loading and destained (Destaining solution: 5% methanol, 7% acetic acid in H_2_O) afterwards. Thereafter, membranes were blocked with 5% skim milk/TBS, followed by incubation with affinity-purified polyclonal rabbit-anti-mouse HDGF antibody solution (dilution 1:1, 000) [7]. Subsequently, membranes were incubated with a goat-anti-rabbit IgG conjugated to horseradish peroxidase (dilution 1:10, 000) (Dianova, Hamburg, Germany). Immunoreactive proteins were visualized with enhanced chemiluminescence (Pierce ECL Western Blotting Substrate, Pierce, Rockford, IL) and exposure to films (Kodak, Stuttgart, Germany) according to manufacturer's instruction.

### Statistical Analysis

Statistical analysis of the epidermoid cysts was carried out by applying a chi-square contingency table test.

## Results

### Generation and genotyping of transgenic mice (HDGF^Tyr^)

**Figure 1:**
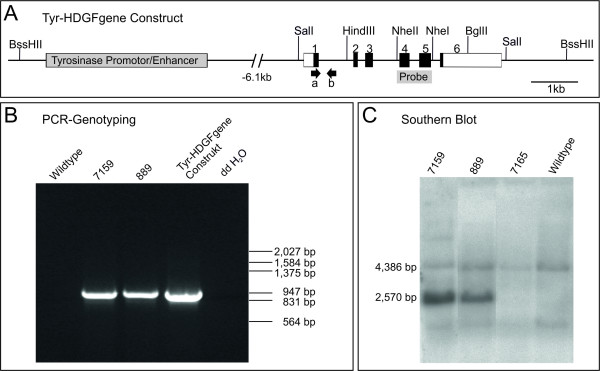
**Generation of HDGF^Tyr ^transgenic mice**. A: Schematic map of the Tyrosinase-HDGFgene construct. The Tyrosinase Promotor/Enhancer element is located approximately 6.1 kb in front of exonI. The HDGF gene consists of the complete exon/intron structure, except intronI was shortened from 6, 377 bp to 786 bp. The construct was separated from the vector backbone by incubation with the restriction enzyme BssHII, purified, and injected into the pronucleus of fertilized mouse egg cells. Boxes 1-6 indicate exons (white parts represent non-coding, black parts indicate coding regions). Arrows show the site of primer annealing for PCR-genotyping. The probe used for Southern blot analysis spans exonIV, intronIV and exonV. B: Examples of a polymerase chain reaction (PCR) genotyping of mouse tail DNA. Primers a and b were used to amplify the 943 bp fragment. Wildtype DNA was used as a negative control. 10 ng of Tyr-HDGFgene construct plasmid DNA was used as a positive control. C: Exemplary illustration of a Southern blot analysis of mouse tail DNA from the PCR-positive offspring 7159 and 889 and the PCR-negative offspring 7165 and wildtype mouse tail DNA as a control. The radioactively labeled probe spanning exonIV, intronIV and exonV hybridizes to both fragments of the wildtype HDGF-allele (4, 386 bp) and the Tyr-HDGFgene construct (2, 570 bp).

To investigate whether HDGF exhibits oncogenic/transforming capacities and whether the observed HDGF-overexpression in various types of tumors promotes tumor development and/or progression, a mouse model was generated overexpressing HDGF only in melanocytes. To allow the expression of possible alternative splicing products we constructed a transgene containing all exons and introns of the murine HDGF gene with a shortened intronI (HDGFgene; Figure 1A). To test the integrity of the protein expressed from this construct it was first cloned under the control of a CMV promoter and expressed in HEK cells. Western blot analysis of cell lysates confirmed the expression of two protein variants at molecular weights resembling HDGF protein variants (Additional file 1A) as can be observed in several tissues [7]. To target the expression to pigment cells, the HDGFgene construct was further cloned under the control of the tyrosinase promoter/enhancer element driving the expression of the melanocyte-specific protein tyrosinase (Figure 1A). Linearized and purified plasmid-DNA was used for pronuclear injection. Out of 61 offspring, 15 were initially genotyped positive for the integration of the Tyr-HDGFgene construct by PCR-genotyping. Figure 1B shows exemplarily the PCR result for the offspring 7159 and 889. In case of an integration of the Tyr-HDGFgene construct, a 943 bp fragment was amplified, which was absent in the wildtype-DNA control. The integration was confirmed by performing a Southern blot (Figure 1C). Due to fragmentation of the genomic DNA with the restriction enzymes BglII and HindIII, two DNA-fragments could be detected with the help of the radioactivly labeled probe encompassing exonIV, intronIV and exonV. The 4, 386 bp fragment, representing the wildtype allele, could be detected in all samples, whereas the 2, 570 bp fragment representing the Tyr-HDGFgene construct was only detected in the samples of the offspring 7159 and 889 but not in the samples of offspring 7165 and the wildtype control. Offspring 7159 and 889 were used as founders for two independent transgenic mouse lines.

### Analysis of HDGF^Tyr ^mice

**Figure 2:**
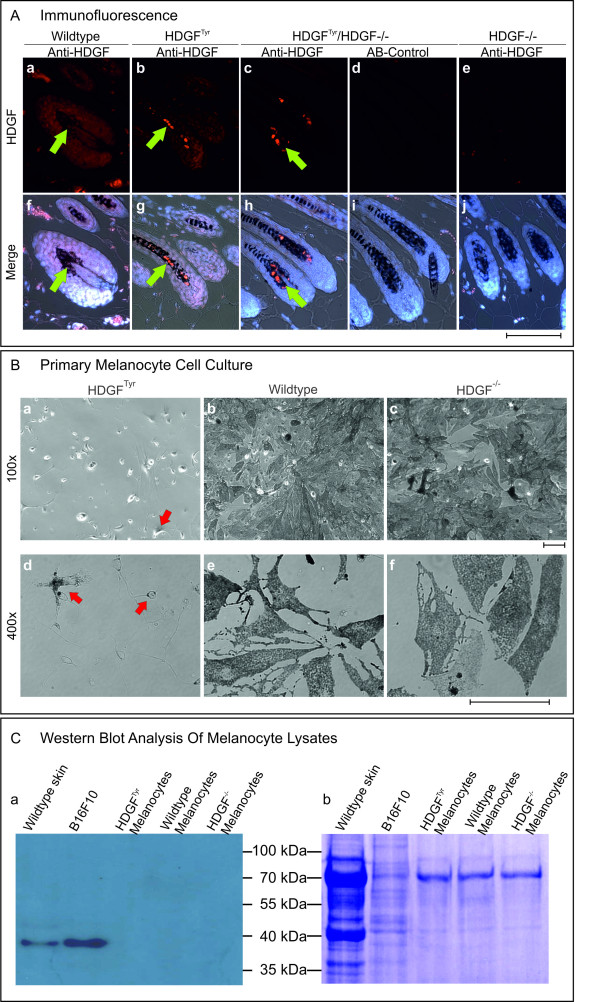
**Detection of melanocyte-specific HDGF-overexpression**. A: Immunofluorescence of skin samples from wildtype, HDGF^Tyr^, HDGF^Tyr^/HDGF^-/- ^and HDGF^-/- ^mice. Paraffin sections were stained with affinity-purified anti-mouse HDGF antibody. Bound antibody was detected by incubation with Cy3 conjugated secondary anti-rabbit antibody. Nuclei were stained with DAPI. a-e HDGF; f-j merge of HDGF, DAPI and transmitted light. Green arrows point at HDGF positive melanocyte nuclei. Scale bar equates 100 μm. B: Primary melanocyte cell culture. Melanocytes were isolated from newborn HDGF^Tyr^, wildtype and HDGF^-/- ^mice. After 2-3 weeks melanocytes from wildtype and HDGF^-/- ^mice grew in a sub-confluent cell layer, whereas very few melanocytes from HDGF^Tyr ^animals (red arrows) were able to survive in cell culture. a-c 100 × magnification; d-f 400 × magnification. Scale bar equates 100 μm. C: Western blot analysis of melanocyte lysates. a HDGF-level of primary melanocytes from HDGF^Tyr^, wildtype and HDGF^-/- ^mice was compared to the level of complete skin and B16F10 (murine melanoma cell line) lysates. 10 μg total protein was loaded onto an SDS-gel. b Equal loading of melanocyte lysate was assessed by staining the membrane with Coomassie solution.

Tyrosinase-HDGF (HDGF^Tyr^) transgenic mice were viable, fertile, and neither showed defects in pigmentation nor did they prematurely turn gray. HDGF expression in skin samples of HDGF^Tyr ^mice was first compared to that of wildtype mice by immunfluorescence (Figure 2A a, b, f, g). Staining of wildtype hair follicles revealed a nuclear HDGF expression in cells of the hair follicle including the pigmented region (Figure 2A a, f). However, in HDGF^Tyr ^mice a bright signal for HDGF was obtained in the pigmented area of the hair follicles (Figure 2A b, g). To ensure that the detected protein represents transgenic HDGF, HDGF^Tyr ^mice were backcrossed into the HDGF^-/- ^mouse line. Indeed, immunfluorescent staining of skin samples from HDGF^Tyr^/HDGF^-/- ^(Figure 2A c, d, h, i) in comparison to HDGF^-/- ^(Figure 2A e, j) mice proofed that the distinct staining pattern seen in HDGF^Tyr ^mice originates from the transcript of the Tyr-HDGFgene construct. *In situ *hybridization using a tyrosinase RNA probe demonstrates that the observed HDGF-staining pattern correlates with the localization of melanocytes in the pigmented area of the hair follicles (Additional file 2). To further investigate the expression of the Tyr-HDGFgene construct, RT-PCRs were performed on cDNA from brain, eye, skin samples (Additional file 1C). The construct was expressed in all three tissues (red arrows) showing strongest expression in the brain. This observation corresponds to an already known leakiness of the tyrosinase promoter in brain tissue [33-36]. Comparison of DNA sequences from the skin PCR-product of wildtype HDGF to transgenic HDGF revealed no mutations over the whole length of the transcript (data not shown). Western blot analysis of skin samples did not reveal significant differences between wildtype and transgenic animals (data not shown) most likely because melanocytes account for only 5-10% of mouse skin cells. Because the RT-PCR results confirmed the previously published leakiness of the Tyrosinase promoter/enhancer construct in the brain, a western blot analysis of brain lysates was performed (Additional file 1B). The result showed that in the brain of HDGF^Tyr^/HDGF^-/- ^mice a HDGF protein could be detected migrating at the same height as HDGF from wildtype tissue (white arrow). To further investigate HDGF expression in melanocytes, primary melanocytes were isolated from neonatal skin of HDGF^Tyr^, wildtype and HDGF^-/- ^mice to quantify the elevated HDGF-expression in HDGF^Tyr ^melanocytes by immunofluorescent stainings. Wildtype and HDGF^-/- ^melanocytes grew in cell culture and differentiated into pigment producing melanocytes while forming a near confluent cell layer (Figure 2B b, c, e, f). Surprisingly, very few pigmented cells could be obtained from HDGF^Tyr ^mice (arrows in Figure 2B a, d). However, Western blot analysis of primary melanocyte lysates revealed that wildtype melanocytes express no substantial amount of HDGF (Figure 2C), whereas HDGF could be detected in B16F10 mouse melanoma cell lysate. In addition HDGF could also not be detected in lysates from HDGF^Tyr ^melanocyte cultures.

In summary, the strong HDGF signal in hair follicle melanocytes from HDGF^Tyr ^and HDGF^Tyr^/HDGF^-/- ^mice compared to wildtype and HDGF^-/- ^ones, respectively as well as the overlapping HDGF-protein and tyrosinase-mRNA expression pattern confirmed that the generation of the HDGF^Tyr ^transgenic mice resulted in an elevated HDGF-expression in hair follicle melanocytes. HDGF^Tyr ^and wildtype mice were monitored over 24 month, but no effect of the additional HDGF could be observed *in vivo*. In particular, no melanocytic lesions or melanoma could be detected in HDGF^Tyr ^mice.

### Analysis of HDGF^Tyr ^mice under tumor growth promoting conditions

**Figure 3:**
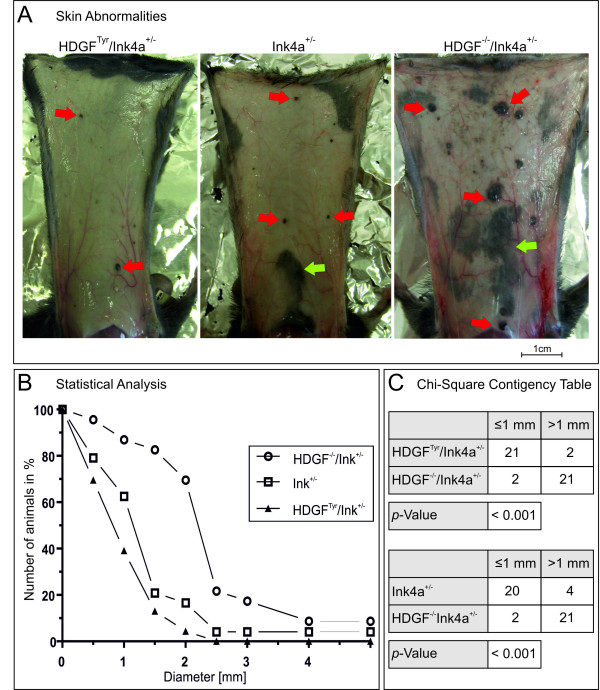
**Statistical analysis of pigmented skin abnormalities (PSA)**. A: Overview of the inner side of skins from HDGF^Tyr^/Ink4a^+/-^, Ink4a^+/-^, and HDGF^-/-^/Ink4a^+/- ^mice showing PSA. Scale bar equates 1 cm. B: Statistical analysis of the occurrence of (PSA) of the different genotypes. HDGF^Tyr^/Ink4a^+/- ^n = 23, Ink4a^+/- ^n = 24; HDGF^-/-^/Ink4a^+/- ^n = 23. C: Chi-square contingency table was applied for the category: largest PSA was ≤ 1mm or > 1mm. Contingency tables were carried out for the following pairs of genotypes: HDGF^Tyr^/Ink4a^+/- ^- HDGF^-/-^/Ink4a^+/- ^and Ink4a^+/- ^- HDGF^-/-^/Ink4a^+/-^. Analysis was regarded significant for p < 0.01.

To uncover the putative oncogenic/transforming capacity of HDGF, HDGF^Tyr ^and HDGF^-/- ^mice were backcrossed with Ink4a^-/- ^mice (kindly provided by Clemens Schmitt (Charité - University of Berlin) with permission of Manuel Serrano [29]). These mice are cancer prone and develop lymphoma and sarcoma spontaneously. Although the Ink4a gene locus is mutated in about 40% of familiar melanoma, Ink4a^-/- ^mice do not develop melanoma spontaneously [29]. But the introduction of an oncogene under the control of a melanocyte-specific promoter like e.g. Tyr-HRas^(G12V) ^[37] or Tyr-Tag [38] results in melanoma development. The emanated HDGF^Tyr^/Ink4a^-/-^, Ink4a^-/- ^and HDGF^-/-^/Ink4a^-/- ^mice were monitored over their reduced life span (5-12 month) and analyzed histologically. Again, the mice were viable, fertile, and showed no altered pigmentation pattern nor did they turn gray prematurely. In addition, no histological alterations concerning melanocyte number or location were observed. Therefore, HDGF^Tyr^/Ink4a^+/-^, Ink4a^+/-^, HDGF^-/-^/Ink4a^+/-^, as well as HDGF^Tyr^, wildtype, and HDGF^-/- ^mice were exposed to an additional mutagenic noxa. For this purpose, mice were neonatally exposed to a single UVB dose of 6.14 kJ/m^2 ^[39,40]. The 1-3 day old mice developed UV-erythema resulting in peeling off the skin after 6-8 days. After eight month animals of the genotypes HDGF^Tyr^/Ink4a^+/-^, Ink4a^+/-^, HDGF^-/-^/Ink4a^+/- ^developed pigmented skin abnormalities (PSA) on tail and back skin, whereas no abnormalities could be observed in HDGF^Tyr^, wildtype, and HDGF^-/- ^mice of up to 12 month after treatment. The PSA of each HDGF^Tyr^/Ink4a^+/-^, Ink4a^+/-^, or HDGF^-/-^/Ink4a^+/- ^mouse were counted, sizes were assessed and statistically evaluated (Figure 3B and 3C), before they were subjected for histological analysis. Figure 3A shows exemplary views of the inner side of the skin of HDGF^Tyr^/Ink4a^+/-^, Ink4a^+/-^, and HDGF^-/-^/Ink4a^+/- ^mice, exposing the highly pigmented lump. The graph shows that HDGF^Tyr^/Ink4a^+/- ^and Ink4a^+/- ^mice developed comparable numbers and sizes of PSA, whereas HDGF^-/-^/Ink4a^+/- ^mice developed both more and larger PSA. These findings were further analyzed by applying a chi-square test to assess if the observed discrepancy was statistically significant (Figure 3C). Accordingly, HDGF^-/-^/Ink4a^+/- ^mice developed significantly more and larger PSA compared to both Ink4a^+/- ^and HDGF^Tyr ^/Ink4a^+/- ^mice (*p *< 0.001). In contrast, the slight tendency of Ink4a^+/- ^mice to develop more PSA compared to HDGF^Tyr ^/Ink4a^+/- ^mice was not statistical significant.

**Figure 4:**
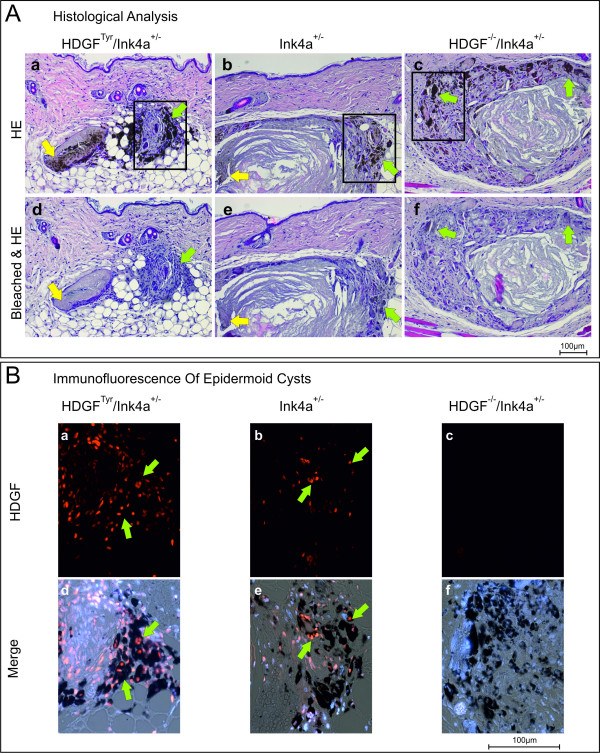
**Histological analysis of pigmented skin abnormalities (PSA)**. A: Serial sections of PSA were either directly HE stained (a-c) or bleached in a benzylalcohol/aceton/H_2_O_2 _based bleaching solution before HE-staining was performed (d-f). Yellow arrows point to the pigmented and to the bleached areas respectively within the cyst lumen; green arrows point to pigmented and bleached melanocytes respectively. B: Immunofluorescence of epidermoid cysts. HDGF was detected by incubation with anti-mouse HDGF antibody and Cy3-conjugated goat-anti-rabbit antibody (a-f). Nuclei were stained with DAPI. a-c HDGF; d-f merge of HDGF, DAPI and transmitted light. Green arrows point to HDGF-positive nuclei in pigmented areas. Scale bars equate 100 μm.

For histological analysis serial sections were HE stained or bleached before HE staining was performed to examine the pigmented areas in detail (Figure 4A). In addition, immunofluorescent staining was performed to asses HDGF-expression in PSA (Figure 4B). HE-stainings revealed that PSA did not resemble melanoma or melanocytic lesions, but rather represent pigmented epidermoid cysts, consisting of a lumen filled with keratinized material surrounded by an epithelial layer of keratinocytes. When comparing the bleached to the non-bleached HE stained sections, it can be noticed that there are only pigmented keratin fibres in the lumen, but no viable cells (yellow arrows Figure 4A). Melanocytes are only located next to the keratinocyte layer (green arrows Figure 4A).

Immunofluorescent staining revealed that both melanocytes and keratinocytes of the epidermoid cysts express HDGF (green arrows Figure 4B a, b, d, e). As expected, no HDGF-expression could be detected in epidermoid cysts derived from HDGF^-/-^/Ink4a^+/- ^mice. Notably, epidermoid cyst associated melanocytes from wildtype animals showed a strong HDGF staining (Figure 4B b), whereas melanocytes in healthy, untreated wildtype hair follicles express HDGF just to a very low extend (Figure 2A a).

In summary, neither melanocyte targeted HDGF-overexpression alone, nor in combination with a second genetic alteration and/or UVB-treatment, led to the development of melanocytic hyperplasia, or lesions in which differences in melanocyte number between the analyzed genotypes could be observed. Interestingly, not HDGF overexpression but HDGF-deficiency led to the development of an increased number and size of epidermoid cysts after UVB-treatment.

## Discussion

HDGF was shown to be overexpressed in several types of tumors, like e.g. hepatocellular carcinoma [17,18] or melanoma [23]. The correlation between the degree of HDGF overexpression, disease prognosis [16,18,20-22,24] and the role for HDGF in tumor angiogenesis [25,26], metastasis [19,26], and apoptosis [27,28] suggested that HDGF may function as an oncogene/protooncogene. In this study, a mouse model was generated to address the question whether HDGF-overexpression mediates oncogenic/transforming capacity to non-transformed cells *in vivo*. We chose to target the HDGF expression to melanocytes with the help of a Tyrosinase promoter/enhancer element (Figure 1A) since results presented by Bernard and co-workers revealed HDGF-expression in melanomas whereas HDGF was absent or weakly present in nontumorigenetic melanocytes [23]. In addition, HDGF-expression was graded with progression, suggesting that the frequency of HDGF-expression increases from benign nevi gradually to late melanoma stages [23]. Therefore, if HDGF exhibits oncogenic/transforming capacity on melanocytes, loss or overexpression of HDGF are expected to have an influence on tumor development and/or progression.

Transgene induced HDGF overexpression in melanocytes could be confirmed by comparing melanocytes and tissues of wildtype and HDGF^Tyr^/HDGF^-/- ^mice (Figure 2, Additional file 1). No alterations regarding melanocyte number and localization could be observed *in vivo *in HDGF^Tyr^, wildtype and HDGF^-/- ^mice, which were additionally analyzed to investigate whether the HDGF-deficiency has an impact on melanocytes in the further conducted experiments. To favor the development of tumors we introduced a second genetic alteration and applied an additional mutagenic noxa. This was done by deleting one copy of the Ink4a tumor suppressor gene and exposure to mutagenic doses of UV-light. Neonatal sunburn of HDGF^Tyr^/Ink4a^+/-^, Ink4a^+/-^, and HDGF^-/-^/Ink4a^+/- ^mice led to the development of pigmented skin abnormalities (Figure 3). These abnormalities, however, did not resemble melanocytic lesions, but strongly resembled human epidermoid cysts. These cysts are benign, round or oval in shape, are in contact with the skin surface via a pore, and are confined by a squamous epithelium which, through cornification, produces a perl of keratinized material [41]. Epidermoid cysts arise most frequently from the infundibulum of hair follicles and represent a keratinocyte differentiation defect. Pigmentation of epidermaoid cysts follows a definite anatomic pattern and is dependent in humans on the natural skin color [42,43]. No differences regarding pigmentation of murine epidermoid cysts of HDGF^Tyr^/Ink4a^+/-^, Ink4a^+/-^, or HDGF^-/-^/Ink4a^+/- ^mice could be observed. Therefore, melanocytes located at the squamous epithelium of epidermoid cysts seem to derive from follicular melanocytes and the observed increased number of melanocytes may be due to growth factors released from cells of the benign cysts. Detailed analysis of size and number of epidermoid cyst per animal revealed a difference between the genotypes. Thus, HDGF^-/-^/Ink4a^+/- ^mice developed significantly more and larger epidermoid cysts compared to both HDGF^Tyr^/Ink4a^+/- ^and Ink4a^+/- ^mice (Figure 3A-C). Morphologically keratinocytes of the outer root sheat (ORS) resemble keratinocytes of the basal cell layer with which they form a continuous cell layer. The morphology of epidermoid cysts indicate that ORS keratinocytes run through the differentiation program of epidermal keratinocytes [44] resulting in the formation of a perl of keratinized material. It is not clear why Ink4a^+/- ^mice develop epidermoid cysts after a single neonatal UVB-treatment, but our results show that additional HDGF-deficiency leads to more and larger epidermoid cysts. The presented results therefore indicate that HDGF in wildtype mice is involved in the control of keratinocyte differentiation. So far, HDGF was reported to promote differentiation of e.g. vascular smooth muscle cells [5,45,46]. In addition, Enomoto and coworkers recently reported on the investigation of an albumin-HDGF transgenic mouse model targeting HDGF-overexpression to hepatocytes [47]. In this case the overexpression of HDGF resulted in delayed hepatocyte maturation, suggesting that HDGF-overexpression in hepatocytes partially suppresses hepatocyte differentiation. In contrast, delayed melanocyte maturation in HDGF^Tyr ^mice was not obvious since skin and hair pigmentation of neonatal HDGF^Tyr ^mice was indistinguishable from wildtype littermates. The only hint on an impact of elevated HDGF-levels on melanocyte differentiation came from *in vitro *experiments. When we tried to culture primary melanocytes from HDGF^Tyr^, wildtype and HDGF^-/- ^newborn mice, HDGF^Tyr ^melanocytes were not able to differentiate and grow in a confluent pigmented cell layer (Figure 2B).

In conclusion, the results obtained here implicate that HDGF does not convey an oncogenic/transforming capacity to melanocytes. In fact, our results and those from other groups point to an involvement of HDGF in cell differentiation. Nevertheless, the overexpression of HDGF in various tumors and its validity as a prognostic marker in some tumors points to a significant role of this growth factor. Over the last years, the concept of non-oncogene addiction (NOA) was established. This concept describes the fact that tumors depend on the expression or overexpression of several proteins, which are not oncogenes, but which are secondarily regulated and thus promote tumor progression [48]. These proteins, like e.g. Stat5 or HSF1, help tumor cells to overcome cellular stress which in normal cells would lead to apoptosis [49,50]. In case of HSF1-deprivation tumor cells die and tumors regress whereas normal cells do not depend on this protein [51]. The latter certainly applies for HDGF as shown by the normal development and health of HDGF^-/- ^mice [15]. On the other hand, *in vitro *and *in vivo *experiments showed that a reduction of HDGF in transformed cells or the injection of HDGF specific antibodies resulted in slowed tumor growth, reduced number of blood vessels, and increased rate of apoptosis [25,52-54]. These results and the results of this study strongly suggest that HDGF does not possess oncogenic/transforming capacities. Instead our results and the results from other groups made us realize that HDGF exhibits many criteria to fit into the concept of non-oncogene addiction. This recommends HDGF as a potential drug target with relevance for a variety of tumors.

## Conclusions

In summary, we developed an animal model overexpressing HDGF in melanocytes. Our results provide no evidence that HDGF has a direct transforming role in tumor development. Instead, the here presented results in combination with previous findings point to a possible role of the growth factor in cell differentiation and suggest that HDGF promotes tumor progression after secondary upregulation. Therefore, HDGF may represent another protein fitting into the concept of non-oncogene addiction of tumor tissue, supporting the assumption of other research groups that HDGF could serve as a target for cancer drug development.

## Competing interests

The authors declare that they have no competing interests.

## Authors' contributions

AS and RG developed the animal models. AS and MA carried out the experiments. NW participated in the statistical analysis of tissue samples. VG participated in study design and drafted the manuscript. SF supervised the study, took part in sample analysis and study design. AS and SF drafted the manuscript. All authors read and approved the final manuscript.

## Pre-publication history

The pre-publication history for this paper can be accessed here:

http://www.biomedcentral.com/1471-2407/11/457/prepub

## Supplementary Material

Additional file 1 **Expressional analysis of the Tyrosinase-HDGFgeneConstruct**. A: Expression of the HDGFgene construct. Lysates of HEK cells transfected with HDGFcdspCDNA3, HDGFgenepBEH or Mock (pBEH) were separated by SDS-gel electrophoresis. HDGF protein expression could be detected in samples transfected with HDGFcdspCDNA3 and HDGFgenepBEH. B: Western blot analysis of brain lysates. Brain lysates from wildtype, HDGF^Tyr^, HDGF^-/- ^and HDGF^Tyr^/HDGF^-/- ^mice were analysed. HDGF could be detected in wildtype, HDGF^Tyr ^and HDGF^Tyr^/HDGF^-/- ^(white arrow) brain lysates. C: Detection of HDGF-mRNA in mouse tissue. Total HDGF-mRNA was detected by performing RT-PCR on cDNA from wildtype and HDGF^Tyr ^tissues using primers spanning the complete HDGF coding sequence (HDGF-sense/HDGF-antisense). The amplificated HDGF-mRNA could be detected as a 841 bp fragment in all samples (lane 1, 3, 5, 7, 9, and 11). Tyr-HDGF-mRNA expressed from the Tyrosinase-HDGFgene construct could only be detected in samples from the transgenic animal (red arrows; 861 bp fragment in lane 4, 8, and 12). Click here for file

Additional file 2 ***In Situ *Hybridisation (ISH) and hematoxylin/eosin (HE) staining of skin samples from HDGF^Tyr^/HDGF^-/-^, HDGF^-/-^, and wildtype mice**. Paraffin sections were used to detect tyrosinase mRNA in melanocytes by incubating the sections with the digoxygenin labeled tyrosinase-antisense (Tyr as) probe or the tyrosinase-sense (Tyr s) probe as a control. Bound probe was detected by incubation with alkaline phosphatase coupled anti-digoxygenin Fab fragment. BM Purple was used as a substrate. a-c HE-staining; d-f ISH Tyr_as; g-i ISH Tyr_s. Scale bar equates 100 μm. Click here for file
