# Supplementary figures and images for: Overexpression of hepatoma-derived growth factor in melanocytes does not lead to oncogenic transformation
Source: BMC Cancer. 2011 Oct 20;11:457. doi: 10.1186/1471-2407-11-457 (PMC3213223; doi:10.1186/1471-2407-11-457)

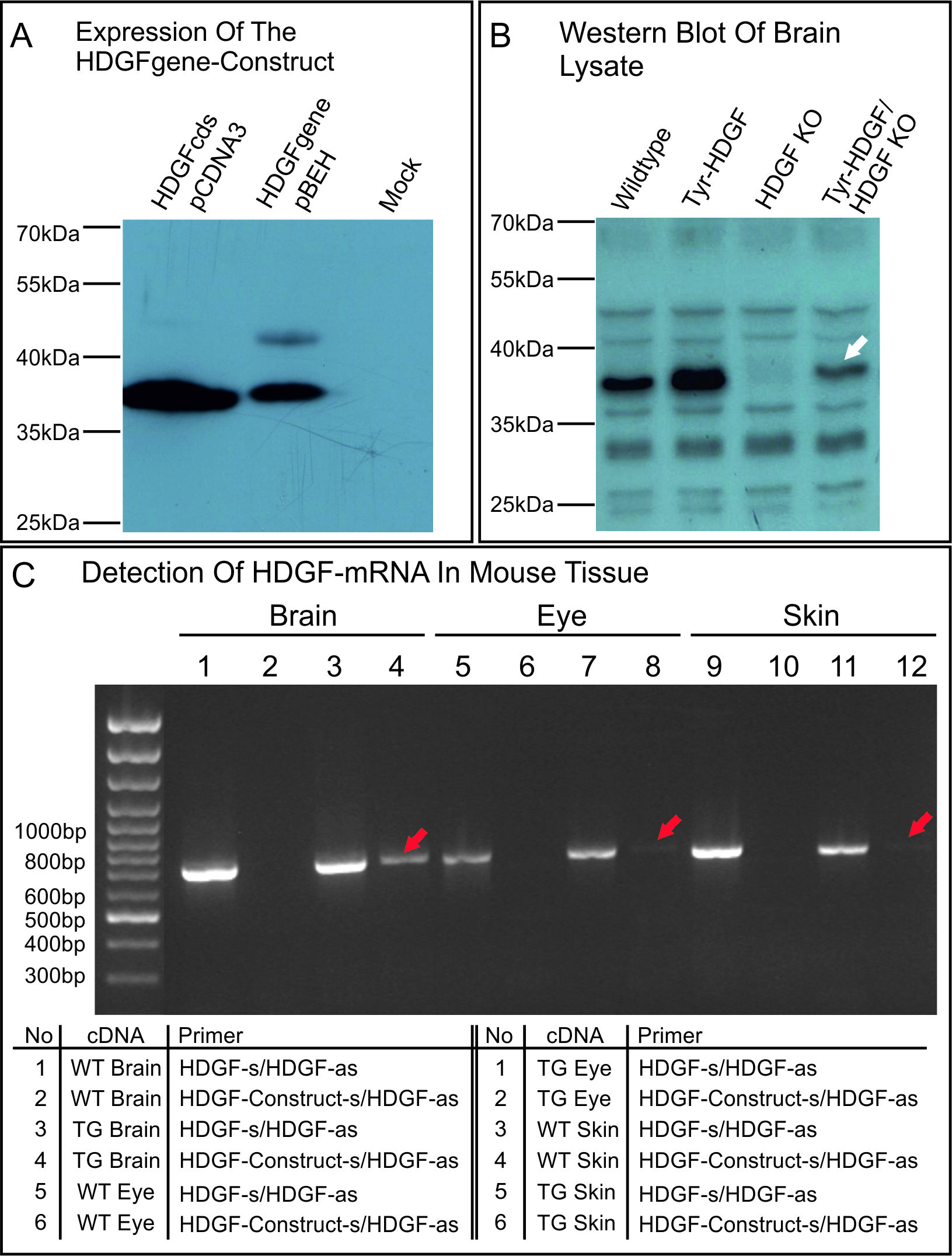

Supplement: Additional file 1 — Expressional analysis of the Tyrosinase-HDGFgeneConstruct. A: Expression of the HDGFgene construct. Lysates of HEK cells transfected with HDGFcdspCDNA3, HDGFgenepBEH or Mock (pBEH) were separated by SDS-gel electrophoresis. HDGF protein expression could be detected in samples transfected with HDGFcdspCDNA3 and HDGFgenepBEH. B: Western blot analysis of brain lysates. Brain lysates from wildtype, HDGFTyr, HDGF-/- and HDGFTyr/HDGF-/- mice were analysed. HDGF could be detected in wildtype, HDGFTyr and HDGFTyr/HDGF-/- (white arrow) brain lysates. C: Detection of HDGF-mRNA in mouse tissue. Total HDGF-mRNA was detected by performing RT-PCR on cDNA from wildtype and HDGFTyr tissues using primers spanning the complete HDGF coding sequence (HDGF-sense/HDGF-antisense). The amplificated HDGF-mRNA could be detected as a 841 bp fragment in all samples (lane 1, 3, 5, 7, 9, and 11). Tyr-HDGF-mRNA expressed from the Tyrosinase-HDGFgene construct could only be detected in samples from the transgenic animal (red arrows; 861 bp fragment in lane 4, 8, and 12). [file 1471-2407-11-457-S1.JPEG]

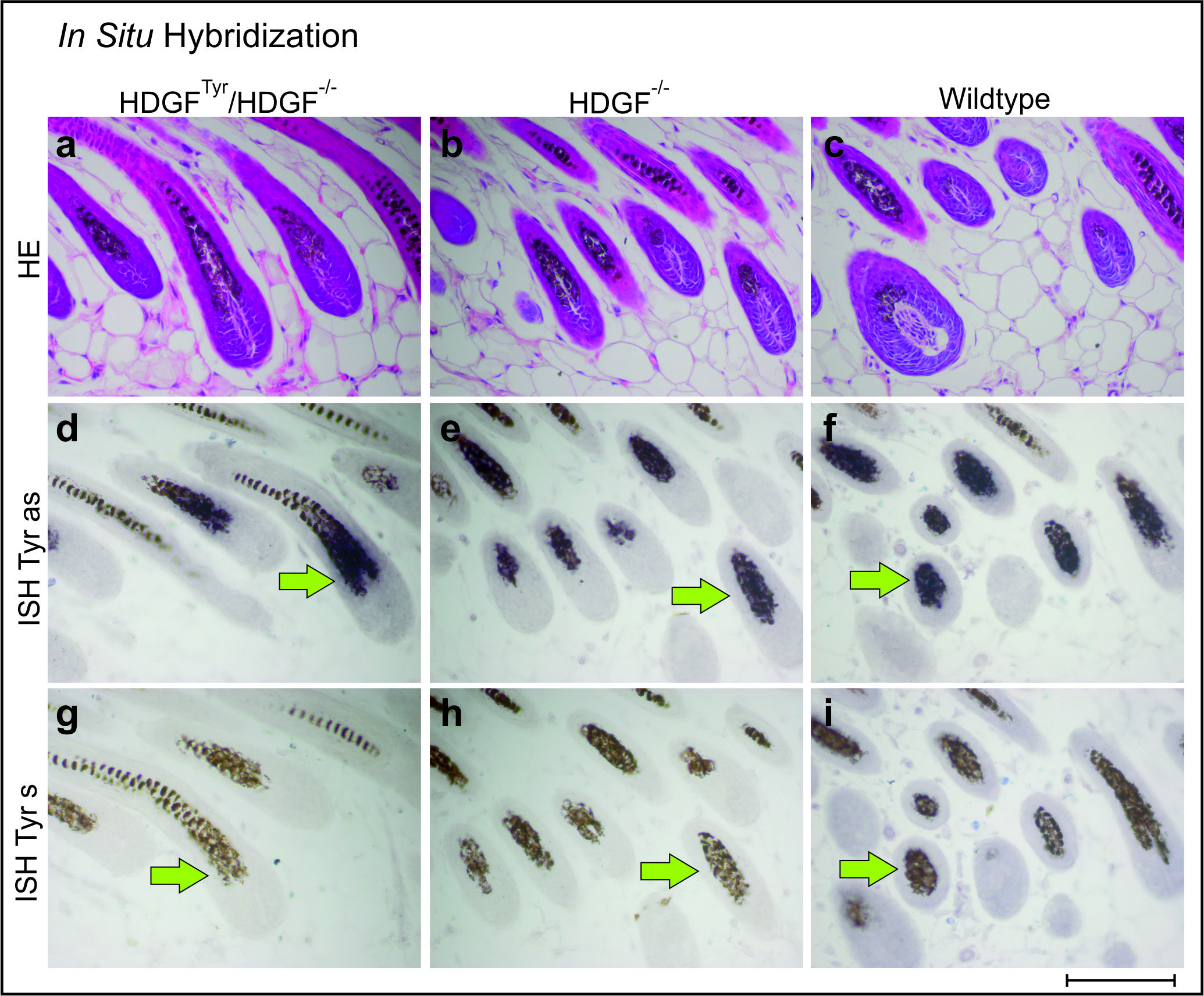

Supplement: Additional file 2 — In Situ Hybridisation (ISH) and hematoxylin/eosin (HE) staining of skin samples from HDGFTyr/HDGF-/-, HDGF-/-, and wildtype mice. Paraffin sections were used to detect tyrosinase mRNA in melanocytes by incubating the sections with the digoxygenin labeled tyrosinase-antisense (Tyr as) probe or the tyrosinase-sense (Tyr s) probe as a control. Bound probe was detected by incubation with alkaline phosphatase coupled anti-digoxygenin Fab fragment. BM Purple was used as a substrate. a-c HE-staining; d-f ISH Tyr_as; g-i ISH Tyr_s. Scale bar equates 100 μm. [file 1471-2407-11-457-S2.JPEG]
